# 3D characterization of walnut morphological traits using X-ray computed tomography

**DOI:** 10.1186/s13007-020-00657-7

**Published:** 2020-08-26

**Authors:** Anthony Bernard, Sherif Hamdy, Laurence Le Corre, Elisabeth Dirlewanger, Fabrice Lheureux

**Affiliations:** 1 Univ. Bordeaux, INRAE, Biologie du Fruit et Pathologie, UMR 1332, 33140 Villenave d’Ornon, France; 2 CTIFL, centre opérationnel de Lanxade, 24130 Prigonrieux, France; 3 GEVES, 49070 Beaucouzé, France

**Keywords:** Walnut, Germplasm collection, Morphological traits, X-ray computed tomography, 3D characterization, Image analysis

## Abstract

**Background:**

Walnuts are grown worldwide in temperate areas and producers are facing an increasing demand. In a climate change context, the industry also needs cultivars that provide fruits of quality. This quality includes satisfactory filling ratio, thicker shell, ease of cracking, smooth shell and round-shaped walnut, and larger nut size. These desirable traits have been analysed so far using calipers or micrometers, but it takes a lot of time and requires the destruction of the sample. A challenge to take up is to develop an accurate, fast and non-destructive method for quality-related and morphometric trait measurements of walnuts, that are used to characterize new cultivars or collections in any germplasm management process.

**Results:**

In this study, we develop a method to measure different morphological traits on several walnuts simultaneously such as morphometric traits (nut length, nut face and profile diameters), traits that previously required opening the nut (shell thickness, kernel volume and filling kernel/nut ratio) and traits that previously were difficult to quantify (shell rugosity, nut sphericity, nut surface area and nut shape). These measurements were obtained from reconstructed 3D images acquired by X-ray computed tomography (CT). A workflow was created including several steps: noise elimination, walnut individualization, properties extraction and quantification of the different parts of the fruit. This method was applied to characterize 50 walnuts of a part of the INRAE walnut germplasm collection made of 161 unique accessions, obtained from the 2018 harvest. Our results indicate that 50 walnuts are sufficient to phenotype the fruit quality of one accession using X-ray CT and to find correlations between the morphometric traits. Our imaging workflow is suitable for any walnut size or shape and provides new and more accurate measurements.

**Conclusions:**

The fast and accurate measurement of quantitative traits is of utmost importance to conduct quantitative genetic analyses or cultivar characterization. Our imaging workflow is well adapted for accurate phenotypic characterization of a various range of traits and could be easily applied to other important nut crops.

## Background

Persian walnut (*Juglans regia* L.), the walnut species cultivated for nut production, is one of the oldest food sources known and is grown worldwide [1]. According to the Food and Agriculture Organization of the United Nations (www.fao.org, 2017 data), worldwide in-shell walnut production exceeds 3.8 M tons. The three largest producers are China, USA and Iran. France is the 9th largest producer, the 2nd in Europe, with 40,000 tons. French walnut orchard area reached approximately 21,000 hectares in 2017 (https://agreste.agriculture.gouv.fr/), making it the most important French fruit crop other than apple. The production is mainly exported in-shell to Europe thanks to its high quality, especially due to the well-known Protected Designations of Origin ‘Noix du Périgord’ and ‘Noix de Grenoble’, the two main walnut production areas. However, the number of cultivars is low and the French walnut industry needs new cultivars well adapted to French climatic conditions, with high nut and kernel quality. The quality traits include round-shaped walnut, smooth and shell easy to crack, larger nut size and high nut/kernel weight ratio [2].

Nowadays, agriculture is facing challenges for crop production and the plant research community needs to perform quantitative analyses of numerous plant traits in order to accelerate progress in breeding [3, 4]. This is why crop germplasm collections are of tremendous importance since no production area is fully self-sufficient in genetic diversity to cover all producer and consumer demand [5]. A germplasm collection is ordinarily evaluated with morphological descriptors which is usually the first step for describing accessions and selecting genitors for breeding programs [6, 7]. For obvious cost reasons, the International Plant Genetic Research Institute (IPGRI, now called Bioversity International) gives ontologies to manage germplasm collections based on morphological measurements, such as fruit length, diameter or thickness in millimeter without any measuring tools mentioned, and visual appreciation for traits that are difficult to quantify such as fruit shape or rugosity.

In walnut, since mid-1970s numerous studies report correlations between various traits related to morphological properties of the nut [8–13]. For instance, to evaluate promising genotypes mainly originated from seed in Iran, shell thickness was measured with a micrometer and shell roughness was recorded by assigning values from 1 to 7 [14, 15] or from 1 to 9 [16, 17] based on visual appreciation recommended by IPGRI [18]. Other works in Iran, using a caliper, also focused on nut length and diameter [19–23]. In Turkey, similar studies were conducted still using a caliper or even a compass to measure walnut diameter, length and/or shell thickness [24–28]. Finally, walnut germplasm collections from Europe were also characterized using similar tools in Albania [29], Serbia [30], Bulgaria [31], Romania [32] and Italy [33].

Overall, the measurements using a caliper, a micrometer or even by simple visual observation are until now the classical methods in walnut assessment. However, this kind of evaluation is painstaking, time-consuming and can lead to inaccuracy and low resolution. Fortunately, imaging techniques applied for plant phenotyping and food quality determination have been developed over the past decades using a wide range of methodologies, mainly for field crops and for various traits such as growth dynamics, shoot structure and morphometric parameters [34]. We can quote the use of visible light [35] and fluorescence imaging [36] on barley; the thermal infrared [37], near-infrared [38] and hyperspectral imaging [39] on rice; or 3D imaging on soybean [40] and magnetic resonance imaging on bean [41]. With the genomics era allowing researchers to unravel the genetic architecture of complex traits, we clearly observe a shift to a need in high-throughput phenotyping for crop improvement [42].

X-ray computed tomography (CT) is a non-destructive imaging technique based on computer-processed X-rays to acquire tomographic slice images of the scanned sample and generate a 3D reconstruction [34]. Used at first for medical purposes, X-ray CT has been lately applied in various agricultural products in particular to evaluate internal quality [43], especially in fruits and vegetables. In apples, X-ray CT was used to evaluate the density and the water content under varying moisture conditions [44], while in pears this technology was used to study the core breakdown development [45]. This method was also used in nuts and few works are reported, such as the detection of pinhole insect damage in almonds [46], the segmentation and classification in hazelnuts [47] and the behaviour study of fourth-instar weevil in pecan nuts [48]. In grapevine, a recent study aimed to characterize inflorescence architecture using X-rays [49]. The authors found correlations between 24 morphological traits among 392 samples of 10 wild *Vitis* species. They were able then to perform a multivariate discriminant analysis to classify the different species.

However, there is not much work showing the application of such methods on walnut. A technical report is available on 3D reconstruction of a walnut using X-ray CT [50] and also a data collection providing an image reconstruction pipeline [51]. However, in this study, the authors aimed to develop a method adapted for machine learning and they used 42 walnuts as models because they have variability, hard shell, softer kernel and empty space which are characteristics similar to the human head. Here, we present the development of a robust method that extracts for the first time complete morphological measurements of walnut using X-ray CT within a worldwide germplasm collection. This method offers the possibility to quantify several traits such as rugosity, sphericity and shape indexes previously really difficult to quantify but essential for French walnut industry. It also allows to evaluate the filling ratio which is the volume occupied by the kernel over the total nut volume, until now impossible to know without cracking the walnut. Our results will be helpful for new breeding programs by selecting the best accessions as genitors in order to release tomorrow’s varieties. Our method could be also used as a reference for walnut or other nut crop germplasm investigation in any breeding program and could pave the way for future application in industry, particularly for internal quality control.

## Methods

### Plant materials and sample preparation

A panel of 161 unique *J. regia* accessions from worldwide was analysed. All the accessions are maintained at the *Prunus* and *Juglans* Genetic Resources Center and located in the Fruit Experimental Unit of INRAE in Toulenne (latitude 44°34′37.442′′N – longitude 0°16′51.48′′W), near Bordeaux, France (Additional file 1). The panel choice was made thanks to a previous work based on genetic diversity and phenotypic variation results [52].

In-shell walnut sampling was performed during harvest season in September 2018 and walnuts were dried following classical French industrial recommendations, for 2 days at 25 °C using a food dryer, and then stored until analyses in a cold room set to 2 °C. For each accession, a selection of 50 walnuts was performed based on their sanitary state and sent to the GEVES laboratory (Beaucouzé, France). All the samples were stored in an environmentally controlled room at 10 °C and 47.75% (± 3.4) relative humidity until use. During the preparation, the walnuts were embedded in a floral foam sample holder (9 length × 8 width × 21 cm height) to keep the samples from any abrupt or slight movement during the scanning process in order to avoid producing distorted images. The floral foam was chosen based on preliminary trials on different low-density materials in order to observe the level of attenuation of the X-rays passing through these materials.

The walnuts were scanned in batches, knowing that, the sample size was not fixed due to the huge variation between the walnuts in size and the limited scanning scope of the detector. The sample size ranged from 5 up to 16 walnuts per scan.

### X-ray computed tomography imaging system specifications

X-ray CT scans were performed at the GEVES laboratory (Beaucouzé, France) using a 3D X-ray imaging system, the NSI X-50 model from North Star Imaging©, Inc. (Minnesota, USA), which has a focus tube with focal spot up to 1 µ, a voltage range of 10–130 kV, an electric current range of 50–300 µA, a flat-panel detector with a resolution of 256 × 256 and an adjustable rotary stage.

### Image acquisition and reconstruction

Scans were obtained at constant electron acceleration energy of 120 kV, an electric current of 300 µA and a rotation speed of 4.99 degrees/s resulting in a scan duration of 14 m34 s. A total of 2,164 images (or radiographs) in a.tif format were used for reconstructing each 3D image using North Star Imaging© reconstruction software EFX-CT (version 1.9.5.12) where the resulting 3D images were exported in a.nsihdr format with a resolution of 992 × 992 × 2991 voxels (voxel size of 0.1 × 0.1 × 0.1 mm).

**Fig. 1:**
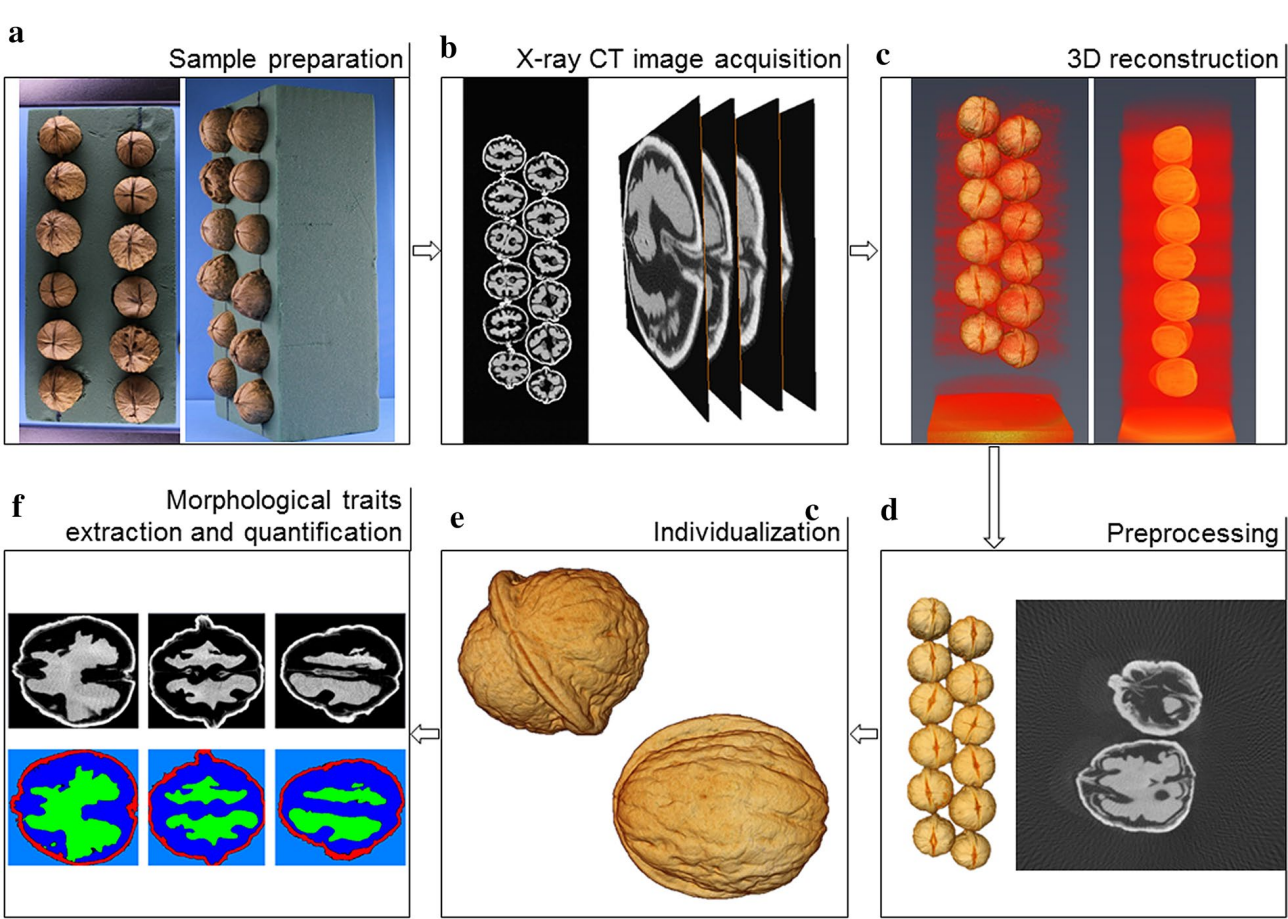
X-ray CT workflow of walnut measurements. **a** Preparation of walnut samples using floral foam (12 walnuts/batch in average), **b** Acquisition of X-ray CT images with right images of 2D slices, **c** 3D reconstruction, **d** Preprocessing with right image showing noise and artifacts that have to be removed, **e** Individualization of each walnut of the batch and **f** XY, YZ, ZX segmentation and labelling of a walnut leading to segmentation of each different part, with the shell in red, the kernel in green and the empty space in blue

**Fig. 2:**
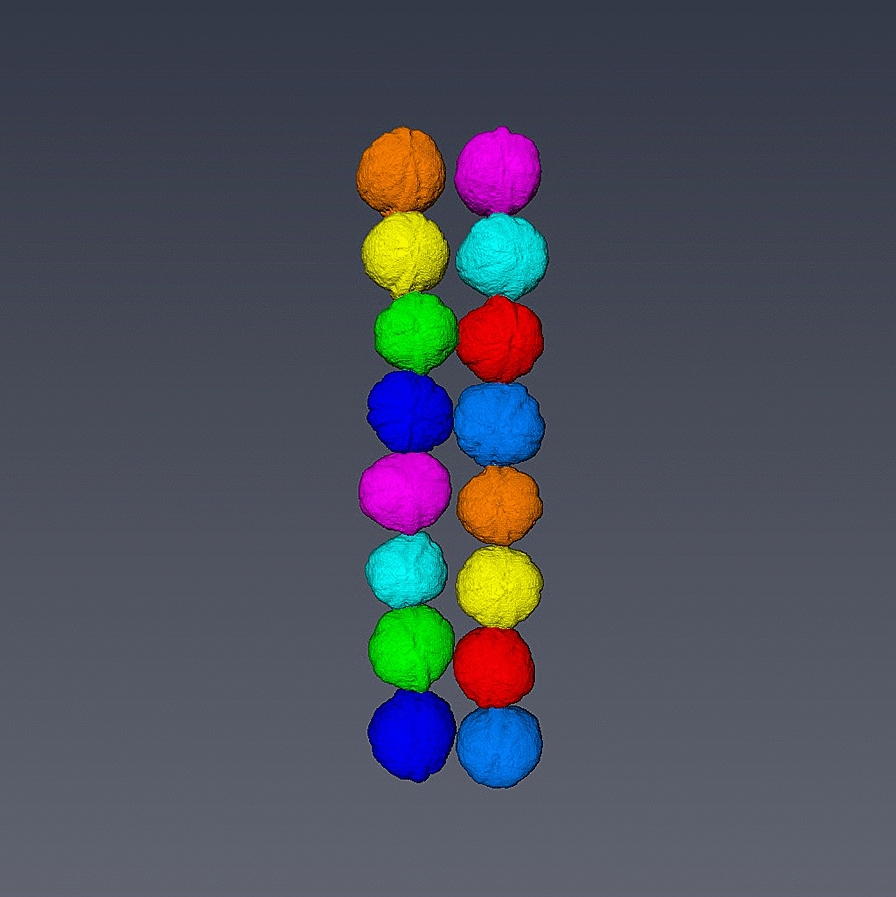
Separation and individualization of walnuts

**Fig. 3:**
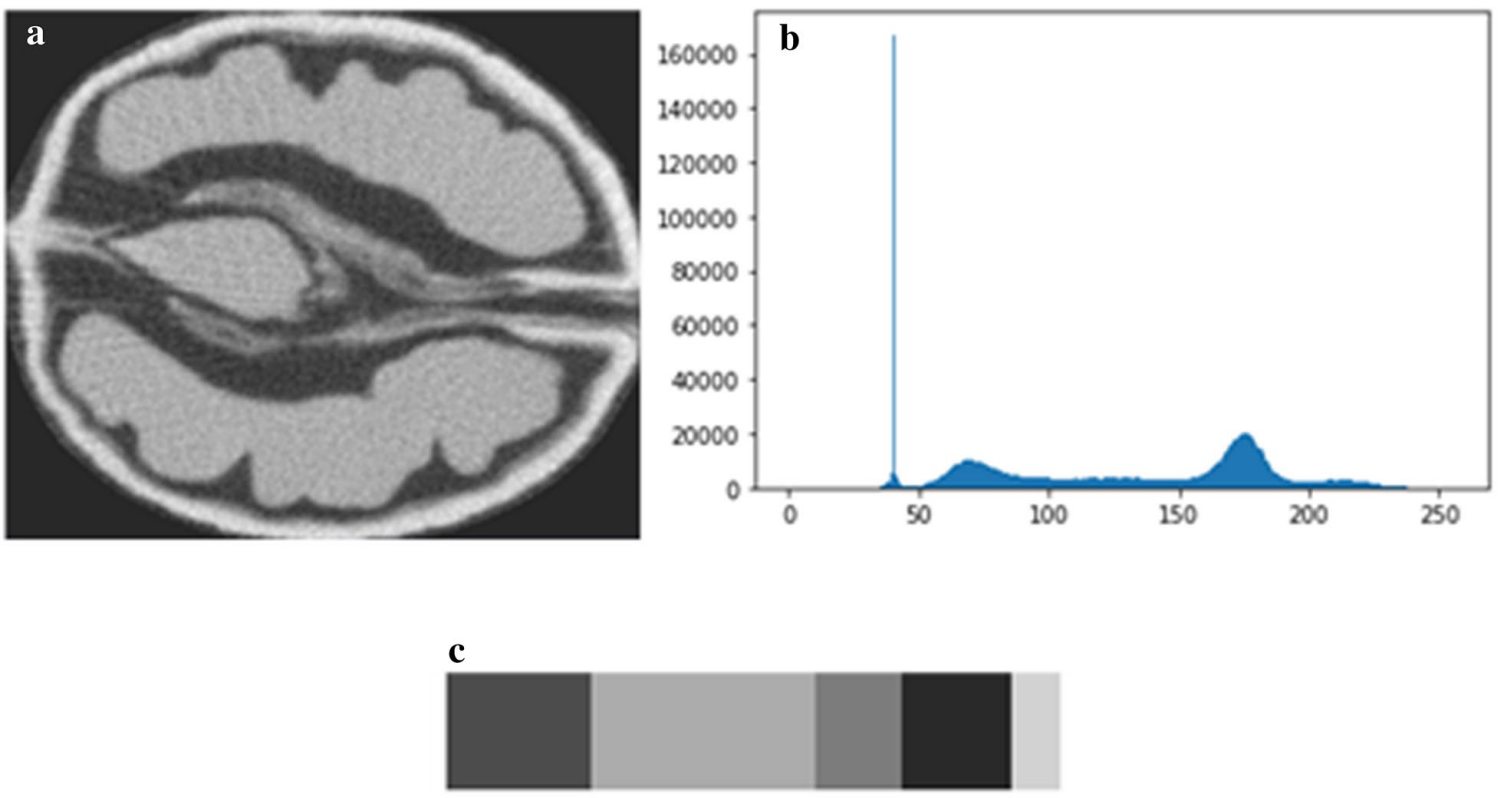
Greyscale histogram analysis. **a** Example of a 2D slice, **b** the corresponding histogram and **c** a bar that visualizes an approximate percentage of pixels in each cluster

After 3D reconstruction, a multi-stage workflow was applied to all CT images in order to eventually achieve a quantitative study. This workflow consists of three key steps as illustrated in Fig. 1d-f: preprocessing steps, walnuts individualization, and morphological traits extraction and quantification.

(*i*) The preprocessing: it begins with automatically loading each image in our image collection ${I}_{(1,x,y,z)},...,{I}_{(m,x,y,z)}$ sequentially (where $m$ is the number of images) and then denoising them in order to eliminate the noise and artifacts introduced by the X-ray system during image acquisition. We applied Gaussian filter, successive morphological operations such as opening and closing, and also, we removed the unwanted small spots in the image. Subsequently, all the voxels which represent the sample holder are eliminated and only the voxels which represent the walnuts are preserved by discarding all the voxels below a certain threshold τ resulting in a binary mask ${M}_{(x,y,z)}$ according to the Eq. (1).

1 $$M_{{\left( {x,y,z} \right)}} = \left\{ {\begin{array}{*{20}c} {0 \,if\,I_{{\left( {x,y,z} \right)}} \le \tau } \\ {1 \,if\,I_{{\left( {x,y,z} \right)}} > \tau } \\ \end{array} } \right.$$

(*ii*) The individualization: the task of extracting the features of the walnuts and quantification is challenging especially if the walnuts are touching. To overcome this difficulty, all the walnuts in the images were separated and individualized (Fig. 2). The individualization step consists of multiple sub-steps such labelling, masking, convex hull estimation and exporting. Labelling is based on voxel connectivity in the whole 3D volume in order to determine the regions of interest which represent the walnuts by assigning identical values to all the voxels that belong to an individual walnut. Each walnut in the image was assigned a unique value starting from 1 to $n$ consecutively where n represents the total number of walnuts in the image. Then, labelling was followed by generating a set of masks using the determined regions of interest then finally, given the original input image and the generated set of masks ${K}_{(x,y,z)}$ as shown in the Eq. (2), each walnut was exported in a separate sub-image ${i}_{(x,y,z)}$ in a.nsihdr format after estimating the convex hull of each walnut. Loading, preprocessing, labelling and convex hull calculation take 20 min for an average sized sample.

2 $$f \left({I}_{(x,y,z)} ,{K}_{(x,y,z)}\right)= \left\{{i}_{(1,x,y,z)} , {i}_{(2,x,y,z)}\dots {i}_{(n,x,y,z)}\right\}$$

(*iii*) The morphological traits extraction and quantification: at this step, the principal components of the walnuts whose morphological features were segmented using multi-level thresholding and watershed algorithm which is a transformation that treats the image like a topographic map [53]. As a consequence, in our case, each main part of each walnut was segmented and was given a unique label as shown in Fig. 1f, considering the kernel, the shell, and the empty space between the kernel and the shell. The optimum threshold τ and greyscale ranges of the principal parts of the walnuts were estimated experimentally based on the analysis of the histogram, using K Nearest Neighbor clustering method, that corresponds to the distribution of the intensities of the images (Fig. 3). Cropping and exporting take 1m30s/walnut. An additional movie file shows this in more detail (Additional file 2).

A fully automated in-house image processing pipeline was developed using the Thermo Scientific Avizo© software V9.0.0 built-in functions, the MATLAB© version 7.7.0 R2008b image processing toolbox [54] from The MathWorks©, Inc. (Massachusetts, USA), the TCL scripting language and Spyder Python IDE. To use this pipeline, walnuts that have no damage on the shell are required.

Measurements of a total of 14 morphological and shape descriptors were obtained: the nut length, the nut face diameter, the nut profile diameter, the nut volume, the nut shape VA3D, the nut Feret shape 3D (defined by D/d where d is the minimum Feret diameter and D is the maximum Feret diameter in the orthogonal direction, so 90° from the minimum Feret diameter; the maximum Feret diameter is the maximum diameter of an object as if it were freely rotating in three dimensions using a caliper [55]), the nut surface area, the shell volume, shell thickness, the kernel volume, the kernel filling ratio, and the empty space volume (Table 1). Quantification takes 2 min/walnut.

**Table 1:** Walnut morphological traits measured by the workflow

Morphological trait	Symbol	Description	Unit
Nut			
Nut length	L	The largest length of the nut from the base to the end	mm
Nut face diameter	F	The largest longitudinal section of the nut through suture	mm
Nut profile diameter	P	The largest longitudinal section of the nut perpendicular to suture	mm
Nut volume	V_n_	Total volume of the nut, V_n_ = V_s_ + V_k_ + V_e_	mm^3^
Nut shape VA3D	S_1_	Shape factor of the nut	–
Nut feret shape 3D	S_2_	Feret shape factor of the nut	–
Nut surface area	A	Surface area of the nut	mm^2^
Nut sphericity	Ψ	Index of nut roundness	–
Shell			
Shell volume	V_s_	Volume of the shell	mm^3^
Shell thickness	T	Thickness of the shell	mm
Shell rugosity	Ω	Index of shell surface roughness	–
Kernel			
Kernel volume	V_k_	Volume of the kernel	mm^3^
Kernel filling ratio	R	Ratio of the kernel volume V_k_ to the total volume of the nut V_n_	%
Empty Space			
Empty space volume	V_e_	Volume of the empty space	mm^3^

In addition, 2 supplementary traits, the nut sphericity (close to roundness) (3) and the shell rugosity (or surface roughness) (4) indexes, were measured using a non-standard calculation since they are not supported by the Avizo© software:

3 $$Nut\,Sphericity = \frac{{\pi^{\frac{1}{3}} \left( {6V_{n} } \right)^{\frac{2}{3}} }}{A}$$

4 $$Shell\,Rugosity=\frac{A}{\sqrt[3]{} 36\pi {{V}_{n}}^{2}}$$

where ${V}_{n}$ is the nut volume and A is its surface area. The sphericity of a sphere is 1 and any object which is not a sphere will have sphericity less than 1. The nut shape VA3D is defined by $\frac{{A}^{3 }}{36\pi {{V}_{n}}^{2}}$.

Experiments were run on a workstation equipped with an Intel® Xeon® dual-core processor running at 3 GHz using 64 MB of RAM and running Windows® version 10. The dataset was assessed using R software [56] with the package “tidyverse” [57]. Pearson correlation matrices were performed using the package “corrplot” [58] and Principal Component Analysis (PCA) using the package “FactoMineR” [59].

## Results

### Building a workflow for assessing phenotypic variation of the germplasm collection from CT images and 3D processing

The X-ray CT imaging workflow permitted to characterize the INRAE germplasm collection for 14 traits whose descriptive statistics (mean, standard deviation, minimum and maximum) are given in Table 2. The collection exhibits high phenotypic variation in morphology-related traits, particularly for Nut Volume which ranged from approx. 10,000 for ‘UK 56–12′ to more than 42,000 mm^3^ for ‘Carmelo’ (Additional file 3). We found between the minimum and the maximum a factor of 1.8 for Nut Length, 1.6 for Nut Face and Nut Profile Diameters, and 2.0 for Shell Thickness. Globally, the Kernel Filling Ratio is low, ranging from approx. 21 to 37%, and the Nut Sphericity has a low variation, from 0.84 to 0.93.

**Table 2:** Descriptive statistics of walnut morphological traits

Morphological trait	Unit	Mean ± SD^a^	Range
Nut			
Nut length	mm	38.39 ± 2.18	28.57–51.43
Nut face diameter	mm	32.27 ± 1.70	25.99–40.75
Nut profile diameter	mm	33.29 ± 1.70	27.06–42.84
Nut volume	mm^3^	19,400.02 ± 2669.03	10,382.05–42,813.08
Nut shape VA3D	–	1.47 ± 0.08	1.24–1.69
Nut feret shape 3D	–	1.25 ± 0.05	1.12–1.48
Nut surface area	mm^2^	4019.53 ± 401.91	2622.59–7093.53
Nut sphericity	–	0.88 ± 0.02	0.84–0.93
Shell			
Shell volume	mm^3^	4076.78 ± 595.05	2390.66–9051.88
Shell thickness	mm	1.03 ± 0.12	0.73–1.49
Shell rugosity	–	1.14 ± 0.02	1.07–1.19
Kernel			
Kernel volume	mm^3^	5723.89 ± 1039.03	3408.85–9548.93
Kernel filling ratio	%	30.02 ± 3.55	20.66–37.42
Empty space			
Empty space volume	mm^3^	9599.35 ± 1719.56	4536.51–24,212.21

^a^SD is the abbreviation for standard deviation

**Fig. 4:**
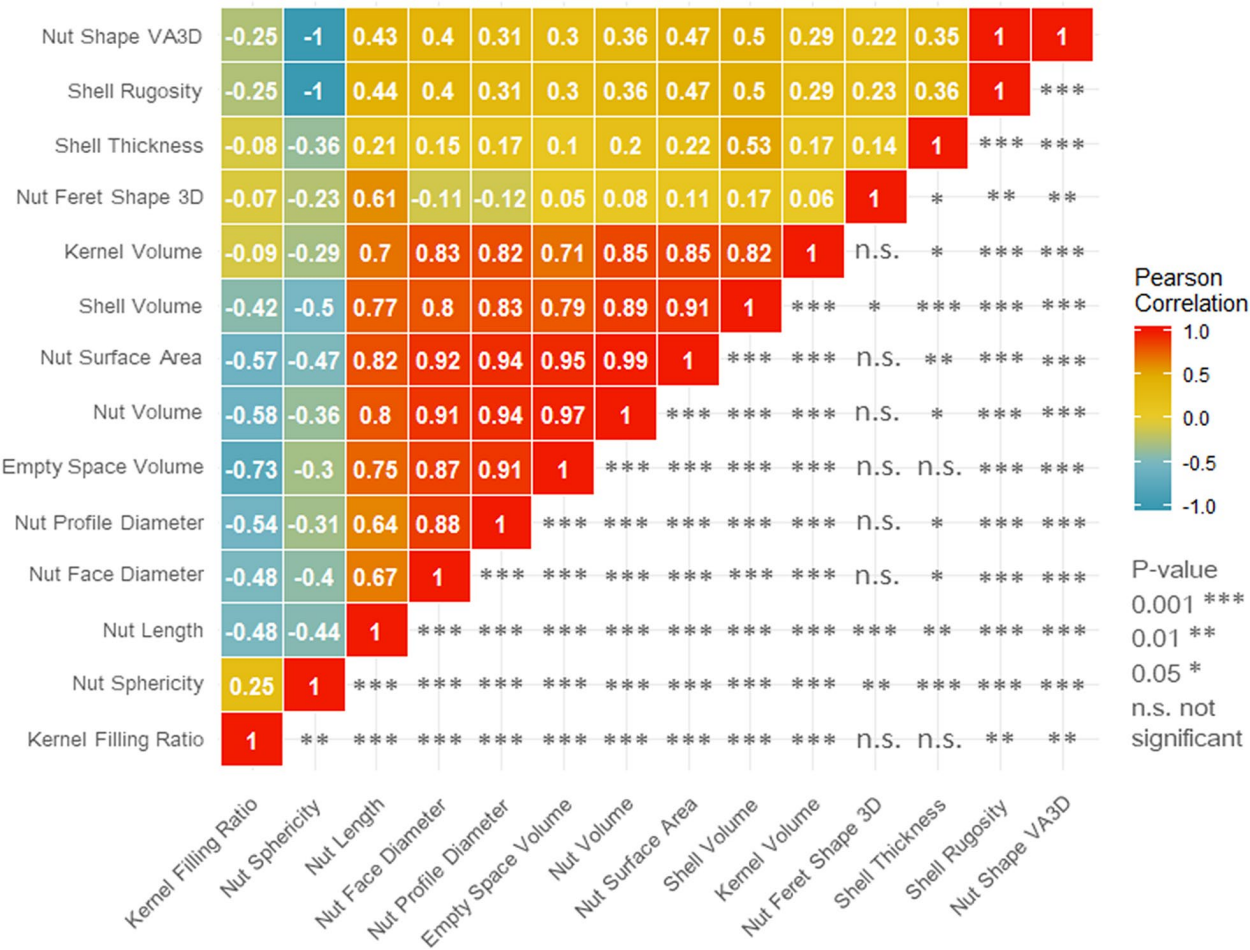
Pearson correlation matrix for walnut morphological traits

Using Pearson correlation coefficient, we found unsurprisingly significant high positive correlations (*p* value 0.001) between all the morphometric traits: Nut Face/Profile Diameters, Nut Length, Nut Surface Area, Nut/Shell/Kernel/Empty Space Volumes (Fig. 4). We can observe that Nut Length is positively correlated with Nut Face and Profile Diameters (0.67 and 0.64, respectively), meaning that the longer the walnut is, the larger the diameter is. Then, Nut Volume is unsurprisingly positively correlated with all three previous traits (Nut Length 0.80, Nut Face Diameter 0.91 and Nut Profile Diameter 0.94), since the variation of the volume of an object depends on those three dimensions. Nut Surface Area is also positively correlated with all volumes-related traits (from 0.85 to 0.99). We also observed significant moderate positive correlations (*p* value 0.001) between all those morphometric traits (except Kernel Volume), and Shell Rugosity and Nut Shape VA3D (from 0.30 to 0.50), indicating that the bigger the fruit is, the rougher the shell is. Those morphometric traits are significantly negatively correlated (*p* value 0.001) with Nut Sphericity and Kernel Filling Ratio. This means that a big fruit is less spherical and less filled by the kernel. Finally, Nut Sphericity is perfectly negatively correlated (*p* value 0.001) with Shell Rugosity and Nut Shape VA3D showing that the closer the fruit gets to a spherical shape, the smoother the shell is.

### The X-ray CT method for the selection of superior genotypes

**Fig. 5:**
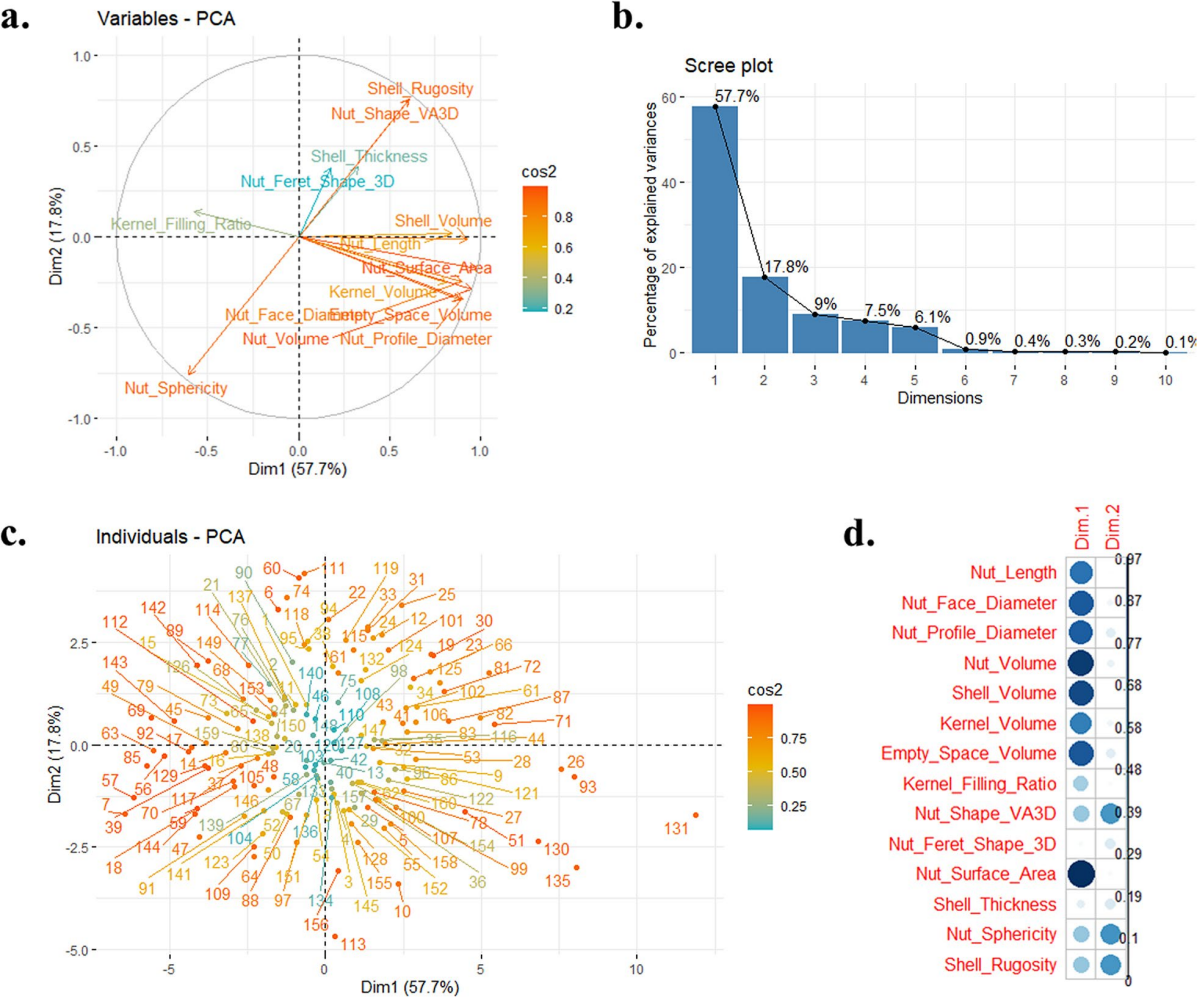
Principal Component Analysis using the 161 walnut accessions and the 14 traits quantified using the X-ray CT method. **a** PCA correlation circle of the 14 variables (dimensions 1 and 2), **b** Scree plot of the percentage of variances explained by the first ten dimensions, **c** PCA scatterplot of the 161 accessions (dimensions 1 and 2), **d** Correlation plot of the 14 variables (dimensions 1 and 2). For a and c plots, color gradient indicates the quality of the representation of each variable given by the squared cosines cos^2^

In French walnut industry, both producers and consumers have particular expectations. For example, they would prefer large round-shaped walnuts, easy to crack and having a high kernel filling ratio. A Principal Component Analysis using the dataset obtained permitted us to select interesting genotypes for the previous important traits (Fig. 5).

The first two dimensions of the PCA explain 75.5% of the total variance (Fig. 5b). The dimension 1 corresponds to the morphometric traits (Nut Length, Nut Face Diameter, Nut Profile Diameter), the volumes (Nut Volume, Shell Volume, Kernel Volume, Empty Space Volume) and the Nut Surface Area (Fig. 5a and d). The dimension 2 is linked to the Nut Shape VA3D, the Nut Sphericity and the Shell Rugosity (Fig. 5a, b). Unfortunately, the Kernel Filling Ratio and the Shell Thickness are both traits badly represented by the PCA (Fig. 5). By visualizing the scatterplot of the individuals, it is now easy to select superior genotypes for most of the traits (Fig. 5c). For example, ‘Carmelo’ (131) and ‘Germisara’ (135) are the two accessions giving the biggest walnuts, and ‘Milotaï n°10′ (113) and ‘Lozeronne n°1′ (7) are the two accessions giving the more round-shaped walnuts.

Since the Shell Thickness and the Kernel Filling Ratio are not well represented by the PCA, we also looked at the ten superior genotypes for both traits (Table 3).

**Table 3:** List of superior genotypes considering the shell thickness and the kernel filling ratio

Superior genotype	Mean ± SD^a^
Shell thickness (mm)	
Lozeronne n°1	0.7316 ± 0.1987
Izvor 10	0.8113 ± 0.1956
H 110–34	0.8293 ± 0.1845
Marchetti	0.8437 ± 0.1855
H 113–21	0.8527 ± 0.0995
Pourpre Hollande	0.8555 ± 0.1752
Sexton	0.8705 ± 0.0270
AS 1	0.8833 ± 0.1520
S 34 B Pyrrus	0.8859 ± 0.1448
H 131–08	0.8865 ± 0.1010
Kernel filling ratio (%)	
IR 13–1	37.42 ± 3.10
H 102–15	35.80 ± 2.77
IR 100–2	35.61 ± 3.20
UK 224–6	35.29 ± 5.53
S 4 B Thétis	35.09 ± 3.59
Lozeronne n°1	35.07 ± 3.03
Wepster W2	34.92 ± 3.19
Ferjean	34.69 ± 3.04
Grappes Suisse	34.51 ± 2.55
Cheinovo	34.37 ± 4.76

^a^SD is the abbreviation for standard deviation

‘Lozeronne n°1′, previously identified as highly round-shaped, gives walnuts with a thin shell, more easily cracked, but, this accession is also one of those that gives the smallest walnuts. The two Iranian accessions ‘IR 13–1′ and ‘IR 100–2′ are among those having the highest kernel/nut ratio. In our panel, although we have a large diversity, we do not find the optimal accession for all the traits, but we can identify the best for each trait with this technique.

## Discussion

Primarily developed for medical purposes, X-ray CT is now widely applied in food science, for instance to track the microstructural evolution of dairy products or to quantify salt concentrations in pork meat [60]. An overview of applications related to various food products showed the use of X-ray CT in chestnut for postharvest assessment of internal decay and in pecan nuts for components screening [61–63]. We showed for the first time that X-ray CT is a method of choice also for walnut germplasm analysis of morphological traits. We obtained quantitative data of high accuracy on 14 traits including volumes and shell thickness which are classically obtained by cracking the nuts. The obtained dataset includes information that is crucial for the INRAE walnut germplasm collection, allowing a precise and relevant characterization of the nuts of each accession. It will help to select superior genitors for a breeding program, so that we can hope to combine many favourable traits in a new variety.

This method is suitable for all types of walnuts, regardless of size and shape. However, the walnuts analysed should not have shell damaged, as, in this case, it is difficult to extract the walnut features with the algorithm developed in our study. The observations show that the shell and the empty space inside those walnuts were incorrectly segmented. The measurements of such walnuts were totally excluded from the results. For future work, it is recommended to discard the damaged walnuts during the sample preparation step, before performing CT scanning in order to avoid any inaccurate calculations. One beneficial direction could be increasing the robustness of the pipeline by designing an artificial intelligence-based task for automatic detection of the walnuts with notably broken and/or damaged shells, and eliminating them before morphological traits extraction and quantification step.

We demonstrate that this method presents numerous advantages compared to classical morphological evaluation, mainly the accuracy of measurement and access to several measures without cracking. For all these reasons, the development of this technology for research scale but also for the industry could be very useful in the future. However, the cost of this technology can constitute a limitation of its use but this method will save time for any lab or industry operator willing to use X-ray CT on walnuts and can be easily transferable to other nuts species. For instance, with the addition of deep learning methods, we can imagine such an application for food security and commercial frauds purposes, in place of molecular biology authentication.

For nuts, it is economically important to know from which cultivar they come from. For French walnut industry, the fruits of the most produced cultivar ‘Franquette’, old cultivar representing 70% of the orchard surfaces, can be distinguished from those of ‘Fernor’, a cultivar released in 1995 and sold at a higher price. Based on our results from 50 walnuts, the value of the nut Feret Shape for ‘Franquette’ is between 1.380 and 1.500 whereas the value for ‘Fernor’ is between 1.210 and 1.304, considering the standard deviation. The Feret shape is clearly a powerful descriptor to discriminate two cultivars and establish genetic origin, since DNA isolation is often a difficult task on nut materials due to the high lipid content of kernels [64].

## Conclusions

We presented a method for better resolution phenotyping of walnuts based on X-ray CT compared to classical measurement methods. The data will be used for INRAE walnut germplasm management, but also for GWAS purposes and for selecting superior genotypes in a future breeding program. This method could be easily adapted for any nut species and potentially moved towards the identification of the first steps of infection by pathogens.

## Supplementary information

**Additional file 1.** List of the 161 accessions studied.

**Additional file 2.** Video of 3D reconstruction and individualization steps of a walnut.

**Additional file 3.** Complete dataset.

## Data Availability

The dataset generated during the current study is available in the Additional file 3.

## Abbreviations

3D: Three-dimensional space
ANR: Agence Nationale de la Recherche
CT: Computed tomography
GEVES: Groupe d’étude et de contrôle des variétés et des semences
GWAS: Genome-wide association study
INRAE: Institut National de Recherche pour l’Agriculture, l’Alimentation et l’Environnement
IPGRI: International Plant Genetic Research Institute
kV: Kilovolt
PCA: Principal Component Analysis
µA: Microamper
